# Mechanism of arrhythmias during the infusion of Ringer’s acetate and Ringer’s lactate solutions during cardiac surgery: new insights

**DOI:** 10.1186/s13054-019-2696-y

**Published:** 2019-12-18

**Authors:** Sébastien Redant, Yael Langman, David De Bels, Rachid Attou, Patrick M. Honore

**Affiliations:** ICU Department, Centre Hospitalier Universitaire Brugmann, Brugmann University Hospital, Place Van Gehuchtenplein, 4, 1020 Brussels, Belgium

We have read with great interest the article by Pfortmueller et al. about fluid management in patients undergoing cardiac surgery [1]. This randomized double blind study showed equivalence between Ringer’s lactate solution and Ringer’s acetate solution in terms of hemodynamic stability, as well as the acid-base and ionic profiles of the two patient populations. However, they observed a higher prevalence of postoperative cardiac arrhythmia in the group receiving Ringer’s lactate solution without a change in the pH or electrolyte values. Previous work has shown that acetate-based dialysate solutions cause hemodynamic and rhythmic disruption. Acetate induces the production of cyclic adenosine monophosphate (cAMP) and cytokines that increase the synthesis of nitric oxide (NO). Studies have shown that acetate-induced NO production induces hypotension during dialysis. Noris et al. showed that the levels of NO and interleukin (IL)-1β are higher after dialysis with acetate than after dialysis with bicarbonate. They suggested that acetate-activated monocytes produce Il-1β that in turn stimulates endothelial cells to produce NO, which can result in hemodynamic instability and arrhythmias [2].

Regarding acid-base balance, it has been shown that Ringer’s lactate solution has a strong ion difference (SID) of 28 while acetate-based solutions have a SID of around 36. Infusion of Ringer’s lactate solution results in a larger reduction in pH when compared to acetate solutions. In vivo, regardless of whether a lactate- or acetate-based solution is infused, serum potassium levels do not change to a degree that could result in rhythm disturbances [3].

Pfortmueller et al. also observed a significant elevation of lactate in Ringer’s lactate solution group compared with Ringer’s acetate solution group (*p* = 0.0065). There is therefore a significant exogenous supply of lactate related to the type of infusion, especially when put into the complex metabolic, hemodynamic, and inflammatory context of cardiac surgery. This exogenous lactate alters the lactate to pyruvate ratio, with the consequent production of glucose at the expense of amino acids in hepatocytes [4]. In addition, the increase in lactate suggests a redox shift, with an increased nicotinamide adenine dinucleotide (reduced and oxidized forms) (NADH/NAD+) ratio in the blood and an increase in cytoplasmic pyruvate. Pyruvate uptake is closely linked to oxidative metabolism which requires adenosine triphosphate (ATP). The fall of ATP induces a leftward shift in the oxygen dissociation curve that can alter oxygen delivery and left ventricular function [5].We believe that these alterations could promote the occurrence of cardiac arrhythmias.

## Data Availability

Not applicable.

## Abbreviations

cAMP: Cyclic adenosine monophosphate
NO: Nitrous oxide
IL-1β: Interleukin-1β
SID: Strong ion difference
ATP: Adenosine triphosphate
NADH/NAD+: Nicotinamide adenine dinucleotide (reduced and oxidized forms)

## References

[1] Pfortmueller CA, Faeh L, Müller M, Eberle B, Jenni H, Zante B (2019). Fluid management in patients undergoing cardiac surgery: effects of an acetate- versus lactate-buffered balanced infusion solution on hemodynamic stability (HEMACETAT). Crit Care.

[2] Noris M, Todeschini M, Casiraghi F, Roccatello D, Martina G, Minetti L (1998). Effect of acetate, bicarbonate dialysis, and acetate-free biofiltration on nitric oxide synthesis: implications for dialysis hypotension. Am J Kidney Dis.

[3] Hofmann-Kiefer KF, Chappell D, Kammerer T, Jacob M, Paptistella M, Conzen P (2012). Influence of an acetate- and a lactate-based balanced infusion solution on acid base physiology and hemodynamics: an observational pilot study. Eur J Med Res.

[4] Barenbrock M, Hausberg M, Matzkies F, de la Motte S, Schaefer RM (2000). Effects of bicarbonate- and lactate-buffered replacement fluids on cardiovascular outcome in CVVH patients. Kidney Int.

[5] Panichi V, Parrini M, Bianchi AM, Andreini B, Cirami C, Finato V (1994). Mechanisms of acid-base homeostasis in acetate and bicarbonate dialysis, lactate hemofiltration and hemodiafiltration. Int J Artif Organs.

